# Exogenous Hydrogen Sulfide Supplementation Alleviates the Salinity-Stress-Mediated Growth Decline in Wheat (*Triticum aestivum* L.) by Modulating Tolerance Mechanisms

**DOI:** 10.3390/plants12193464

**Published:** 2023-10-02

**Authors:** Khalid H. Alamer

**Affiliations:** Biological Sciences Department, Faculty of Science and Arts, King Abdulaziz University, Rabigh 21911, Saudi Arabia; kalamer@kau.edu.sa

**Keywords:** antioxidants, glycine betaine, glyoxylase I, hydrogen sulfide, proline, oxidative stress

## Abstract

The impact of the exogenous supplementation of hydrogen sulfide (20 and 50 µM HS) on growth, enzyme activity, chlorophyll pigments, and tolerance mechanisms was studied in salinity-stressed (100 mM NaCl) wheat. Salinity significantly reduced height, fresh and dry weight, chlorophyll, and carotenoids. However, the supplementation of HS (at both concentrations) increased these attributes and also mitigated the decline to a considerable extent. The exogenous supplementation of HS reduced the accumulation of hydrogen peroxide (H_2_O_2_) and methylglyoxal (MG), thereby reducing lipid peroxidation and increasing the membrane stability index (MSI). Salinity stress increased H_2_O_2_, MG, and lipid peroxidation while reducing the MSI. The activity of nitrate reductase was reduced due to NaCl. However, the supplementation of HS alleviated the decline with obvious effects being seen due to 50 µM HS. The activity of antioxidant enzymes (superoxide dismutase, catalase, ascorbate peroxidase, and glutathione reductase) was assayed and the content of reduced glutathione (GSH) increased due to salt stress and the supplementation of HS further enhanced their activity. A decline in ascorbic acid due to salinity stress was alleviated due to HS treatment. HS treatment increased the endogenous concentration of HS and nitric oxide (NO) under normal conditions. However, under salinity stress, HS supplementation resulted in a reduction in HS and NO as compared to NaCl-treated plants. In addition, proline and glycine betaine increased due to HS supplementation. HS treatment reduced sodium levels, while the increase in potassium justified the beneficial role of applied HS in improving salt tolerance in wheat.

## 1. Introduction

Salinity is a global problem and has been considered to be one of the main threats to sustainable food production. Salinity stress results from the excess accumulation of toxic ions within the soil, which hampers the growth of roots, restricts access to essential mineral ions, and reduces water uptake, hence affecting the overall developmental events [1]. Salt stress significantly affects germination, root and shoot growth, photosynthesis, enzyme activity, and yield [2,3]. It has been reported that salinity considerably declines photosynthesis, enzyme functioning, redox homeostasis, and the uptake of key mineral ions, resulting in growth and yield restrictions [4]. Harmful effects of salt stress are associated with an increased accumulation of reactive oxygen species, including hydrogen peroxide, hydroxyl, superoxide, and singlet oxygen, which reveal modifications in the structural and functional integrity of proteins, lipids, etc. [5]. Such oxidative effects of stresses hamper cellular functioning, causing significant alterations in the metabolism and adaptability of plants to withstand adverse conditions [6]. Naturally occurring tolerance mechanisms are induced when plants sense the excess accumulation of toxic radicals. Antioxidant systems, glyoxylase systems, osmolytes, and secondary metabolites form key components of the tolerance mechanisms against the stresses [5,6,7]. It has been reported that the upregulation of these tolerance mechanisms provides stability to growth, photosynthesis, and enzyme functioning, and hence contributes to yield enhancement [8]. The upregulation of antioxidants [9,10], glyoxalases [7,11], and osmolytes [8], as well as the sequestering and compartmentalization of toxic ions [4], determines the potential of plant species to withstand the adversaries of salinity. From stress sensing to the elicitation of response, several molecules have key roles. Continuous efforts are being made to improve the tolerance of plants to stresses.

Hydrogen sulfide (HS) is a bioactive gas and is considered to be a gas transmitter in animals associated with several health benefits [12]. However, recent research has witnessed its involvement in plants as well. Studies have shown that HS improves germination, growth, photosynthesis, and tolerance to stresses in plants [13,14]. It has been reported that the beneficial effects of HS in plant stress tolerance are caused by its effect on indigenously occurring mechanisms and the crosstalk with other signaling molecules to drive adaptability [15]. Wang et al. [16] have demonstrated that HS mediates salinity tolerance in *Medicago sativa* by modulating nitric oxide. Hydrogen sulfide activates SOS pathway to lower the accumulation of Na in wheat [17]. In addition, HS regulates the redox network to prevent protein degradation via post-translational modifications [15,18]. Increased tolerance to arsenic [19], cadmium [20], drought [21], salinity [13], and heat [8] stress due to HS treatments has been attributed to the upregulation of tolerance mechanisms.

Wheat is an important cereal crop grown widely throughout the world and provides food for the majority of world’s population. It is rich in carbohydrates, proteins, fiber, etc. However, salinity stress considerably affects its growth and yield quality. Therefore, this study was performed to evaluate the beneficial role of the exogenous supplementation of HS in protecting wheat from the adverse effects of salinity stress. It was hypothesized that exogenous HS supplementation can protect wheat against salinity by influencing tolerance mechanisms, like antioxidant systems, glyoxylase activity, and osmo-protectant accumulation.

## 2. Results

### 2.1. Effect of HS on the Height, Fresh, and Dry Weight of Shoot

**Figure 1:**
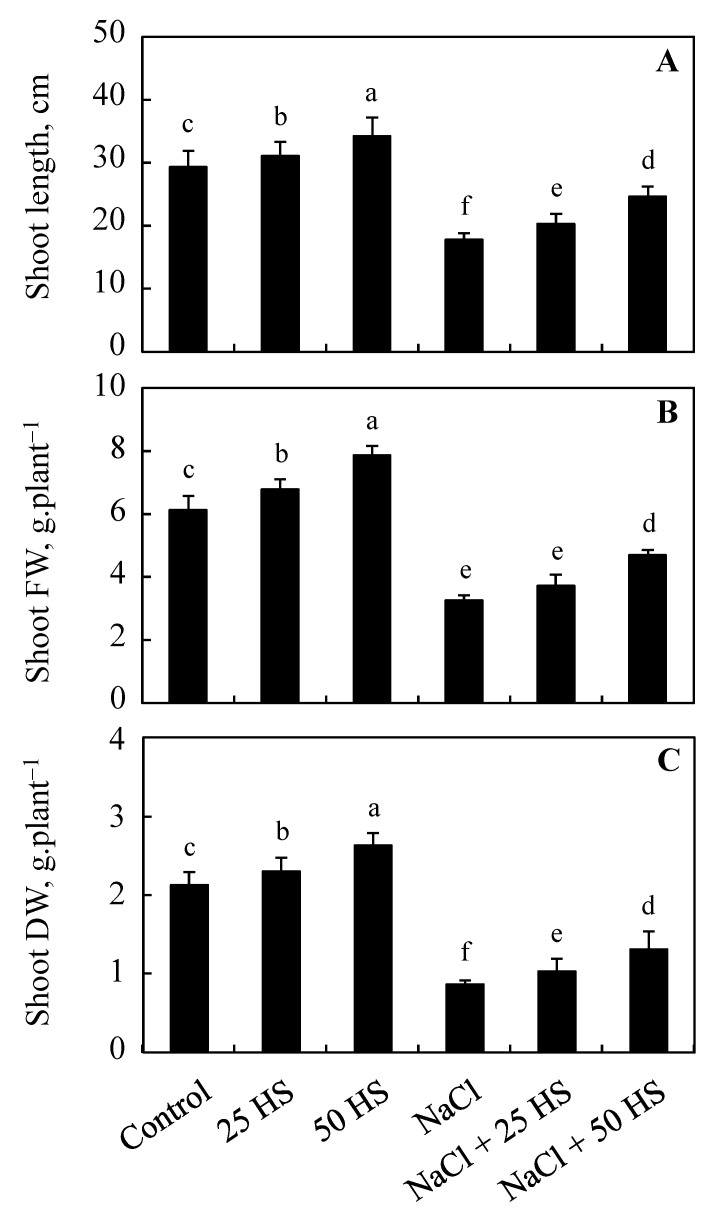
Effect of the exogenous supplementation of hydrogen sulfide (20 and 50 µM HS) on the (**A**) shoot length, (**B**) shoot fresh weight, and (**C**) shoot dry weight of wheat (*Tritium aestivum* L.) under salinity (100 mM NaCl) stress. The mean (±SE) of three replicates is given and bars with different letters are significantly different at *p* < 0.05.

Salinity stress resulted in significant reductions in the shoot height, as well as the fresh and dry weight, of wheat plants. Relative to the control, the height, fresh weight, and dry weight declined by 39.31%, 46.98%, and 59.27%, respectively. However, HS treatment at both concentrations caused an enhancement in these parameters and also alleviated the decline caused due to NaCl. The height increased by 17.83%, the fresh weight increased by 28.22%, and the dry weight increased by 24.05% due to the 50 µM HS over the control. Relative to the control, the height, shoot fresh weight, and shoot dry weight of NaCl + 50 µM HS decreased by 15.92%, 23.49%, and 38.01%, respectively (Figure 1A–C).

### 2.2. Effect of Exogenous HS on Photosynthetic Pigments

**Figure 2:**
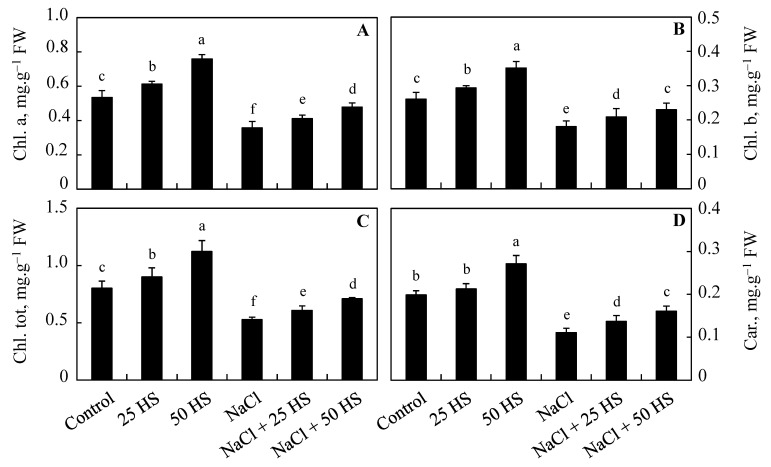
Effect of the exogenous supplementation of hydrogen sulfide (20 and 50 µM HS) on (**A**) chlorophyll a, (**B**) chlorophyll b, (**C**) total chlorophyll, and (**D**) carotenoids of wheat (*Tritium aestivum* L.) under salinity (100 mM NaCl) stress. The mean (±SE) of three replicates is given and bars with different letters are significantly different at *p* < 0.05.

HS treatment, at both concentrations, increased the contents of photosynthetic pigments over the control (Figure 2). However, salinity caused a considerable decline in chlorophyll a (33.09%), chlorophyll b (30.71%), total chlorophyll (34.20%), and carotenoids (44.00%) over the control plants. HS treatment applied to NaCl-stressed plants considerably mitigated the decline with much evident mitigation observed in plants grown with NaCl + 50 µM HS. Relative to the control, declines of only 10.51% (chlorophyll a), 11.92% (chlorophyll b), 11.48% (total chlorophyll), and 18.90% (carotenoids) were observed in plants treated with NaCl + 50 µM HS. Under normal growth conditions, increases of 42.01% (chlorophyll a), 34.62% (chlorophyll b), 39.70% (total chlorophyll), and 36.79% (carotenoids) were observed in plants treated with 50 µM HS over the control (Figure 2A–D).

### 2.3. The Impact of HS Treatment and NaCl Stress on Oxidative Stress Parameters

**Figure 3:**
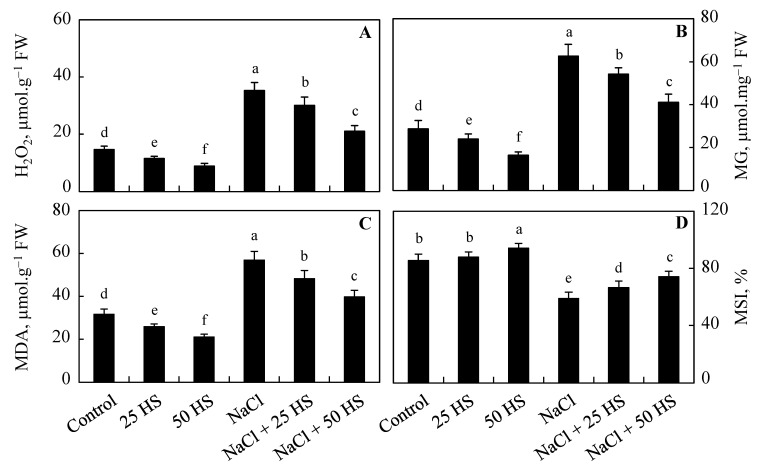
Effect of the exogenous supplementation of hydrogen sulfide (20 and 50 µM HS) on the (**A**) hydrogen peroxide (H_2_O_2_), (**B**) methylglyoxal (MG), (**C**) lipid peroxidation (MDA), and (**D**) membrane stability index (MSI) of wheat (*Tritium aestivum* L.) under salinity (100 mM NaCl) stress. The mean (±SE) of three replicates is given and bars with different letters are significantly different at *p* < 0.05.

Results on oxidative stress parameters, including hydrogen peroxide (H_2_O_2_), lipid peroxidation (measured as malonaldehyde (MDA) content and methylglyoxal (MG) content), and the membrane stability index (MSI), are shown in Figure 3. Salinity (100 mM NaCl) increased the concentration of H_2_O_2_ (139.56%), MG (118.23%), and MDA (79.72%), causing a decline in the MSI (31.12%) over the control plants (Figure 3). Exogenous HS treatment at both concentrations caused a decline in oxidative stress parameters, reflecting the increased MSI. Relative to the control, due to 20 µM HS treatment, H_2_O_2_, MG, and MDA decreased by 20.32%, 16.01%, and 18.31%, and, due to 50 µM HS treatment, they decreased by 39.56%, 42.70%, and 33.25%. Treatment with 50 µM HS dramatically alleviated the salinity-stress-induced oxidative stress by causing a decline of 40.24% in H_2_O_2_, 34.35% in MG, and 30.12% in MDA, resulting in an enhancement of 25.98% in the MSI over the NaCl-stressed plants (Figure 3A–D).

### 2.4. Effect of HS on Proline and Glycine Betaine

**Figure 4:**
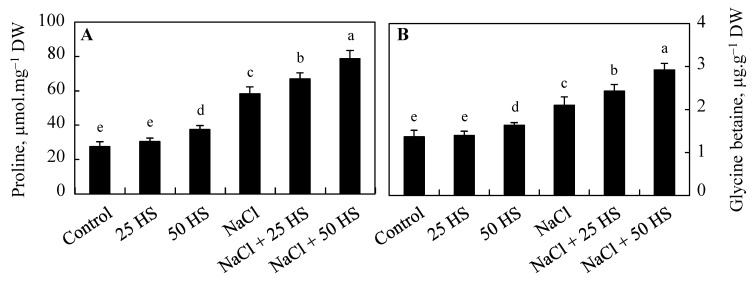
Effect of the exogenous supplementation of hydrogen sulfide (20 and 50 µM HS) on the (**A**) proline and (**B**) glycine betaine of wheat (*Tritium aestivum* L.) under salinity (100 mM NaCl) stress. Data are the mean (±SE) of three replicates and letters on bars show significant differences at *p* < 0.05.

Proline and glycine betaine content caused increases of 112.16% and 54.41%, respectively, over the control due to NaCl stress. HS treatment at both concentrations caused a further increase in proline and glycine betaine content over the control, attaining dramatic increases of 186.45% and 114.20% in NaCl + 50 µM HS over the control. Under normal conditions, relative to the control, proline and glycine betaine content increased by 10.45% and 2.94%, respectively, due to 20 µM HS and by 36.35% and 19.76%, respectively, due to 50 µM HS (Figure 4A,B).

### 2.5. Effect of HS and NaCl Stress on the Antioxidant System

**Figure 5:**
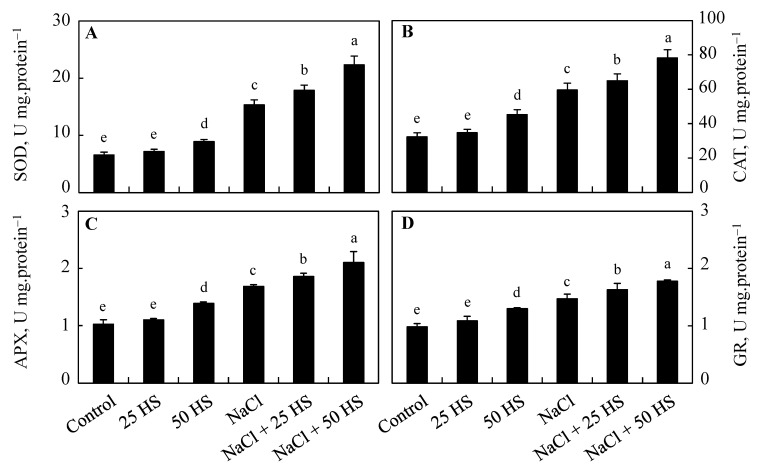
Effect of the exogenous supplementation of hydrogen sulfide (20 and 50 µM HS) on the (**A**) superoxide dismutase (SOD), (**B**) catalase (CAT), (**C**) ascorbate peroxidase (APX), and (**D**) glutathione reductase (GR) activity of wheat (*Tritium aestivum* L.) under salinity (100 mM NaCl) stress. The mean (±SE) of three replicates is given and bars with different letters are significantly different at *p* < 0.05.

Plants grown under salinity stress showed a significant increase in antioxidant enzyme activities over the control. Exogenous HS treatment also increased their activity compared to the control and caused further increases when applied in combination with NaCl. Under normal conditions, relative to the control, increases of 9.80%, 7.35%, 6.66%, and 9.53% were observed for superoxide dismutase (SOD), catalase (CAT), ascorbate peroxidase (APX), and glutathione reductase (GR), respectively, due to 20 µM HS treatment, while increases of 18.92%, 15.04%, 15.89%, and 31.54% were observed following 50 µM HS. Relative to the control, the activity of antioxidant enzymes dramatically increased in plants treated with NaCl + 50 µM HS. Percentage increases of 45.66% (SOD), 31.41% (CAT), 31.25% (APX), and 20.40% (GR) were observed in plants treated with NaCl + 50 µM HS over NaCl-treated plants (Figure 5). 

**Figure 6:**
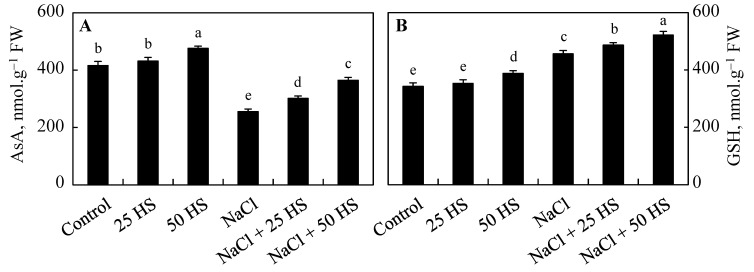
Effect of the exogenous supplementation of hydrogen sulfide (20 and 50 µM HS) on the (**A**) ascorbic acid (AsA) and (**B**) reduced glutathione (GSH) content of wheat (*Tritium aestivum* L.) under salinity (100 mM NaCl) stress. The mean (±SE) of three replicates is given and bars with different letters are significantly different at *p* < 0.05.

Salinity stress resulted in a 18.39% reduction in ascorbic acid (AsA). However, this increase caused a 28.45% reduction in glutathione (GSH) over the control. HS treatment increased both ascorbic acid and GSH over the control, following dramatic increases of 9.43% and 7.88%, respectively, as a result of 50 µM HS treatment. Increases of 4.21% and 16.50% were observed in ascorbic acid over NaCl-treated plants due to 20 µM HS and 50 µM HS treatments. GSH content dramatically increased by 47.12% in plants treated with NaCl + 50 µM HS contrary to the control (Figure 6).

### 2.6. Influence of HS and NaCl on Glyoxylase I Activity

**Figure 7:**
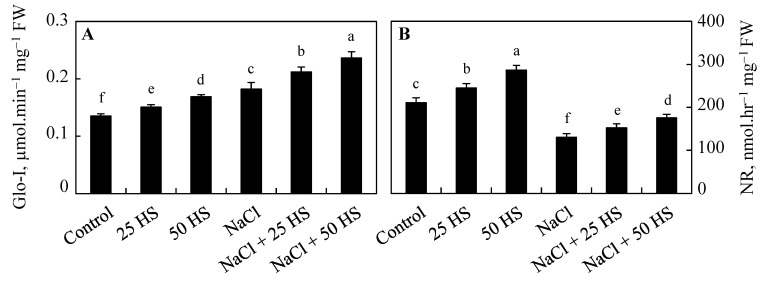
Effect of the exogenous supplementation of hydrogen sulfide (20 and 50 µM HS) on the (**A**) glyoxylase I (Glo-I) and (**B**) nitrate reductase (NR) activity of wheat (*Tritium aestivum* L.) under salinity (100 mM NaCl) stress. The mean (±SE) of three replicates is given and bars with different letters are significantly different at *p* < 0.05.

Salinity stress induced the activity of glyoxylase I over the control and treatment of HS, causing further dramatic increases due to 50 µM HS. Relative to the control, the activity of glyoxylase I increased by 5.80% and 14.83% due to 20 µM and 50 µM HS, respectively, under no-stress conditions. A percentage increase of 29.48% was observed in the activity of glyoxylase I due to NaCl stress. HS treatment applied to NaCl caused further increases of 10.06% and 32.83% in NaCl + 20 µM HS and NaCl + 50 µM HS, respectively, over NaCl-stressed counterparts (Figure 7A).

### 2.7. Effect of HS and NaCl on Nitrate Reductase Activity

Due to NaCl, the activity of nitrate reductase exhibited a significant decline (62.00%) over the control; however, relative to the control, HS treatment increased its activity by 16.66% at 20 µM concentrations and by 36.19% at 50 µM concentrations. The supplementation of HS to NaCl-treated plants resulted in a significantly bigger decline. In comparison to NaCl-stressed plants, the activity of nitrate reductase dramatically declined by 34.53% in plants treated with NaCl + 50 µM HS (Figure 7B).

### 2.8. Effect of HS and NaCl on the Content of Phenol and Activity of Phenylalanine Ammonia-Lyase

**Figure 8:**
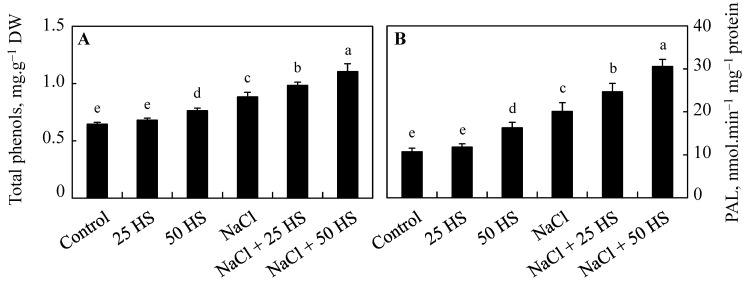
Effect of the exogenous supplementation of hydrogen sulfide (20 and 50 µM HS) on (**A**) the endogenous content of total phenols and (**B**) the activity of phenylalanine ammonia-lyase (PAL) in wheat (*Tritium aestivum* L.) under salinity (100 mM NaCl) stress. The mean (±SE) of three replicates is given and bars with different letters are significantly different at *p* < 0.05.

HS treatment at both concentrations significantly increased the content of phenols and the activity of phenyl alanine ammonia lyase over control and NaCl-stressed plants. Relative to the control, an increase of 37.02% was observed in the content of phenols and an increase of 88.27% in the activity of phenylalanine ammonia-lyase was observed due to NaCl. HS treatment using NaCl-stressed counterparts resulted in further dramatic increases of 24.75% and 51.96%, respectively, in phenol and phenylalanine ammonia-lyase activity over NaCl-treated plants following NaCl + 50 µM HS treatment (Figure 8A,B).

### 2.9. Influence of NaCl and HS on the Content of Sodium (Na) and Potasssium (K)

**Figure 9:**
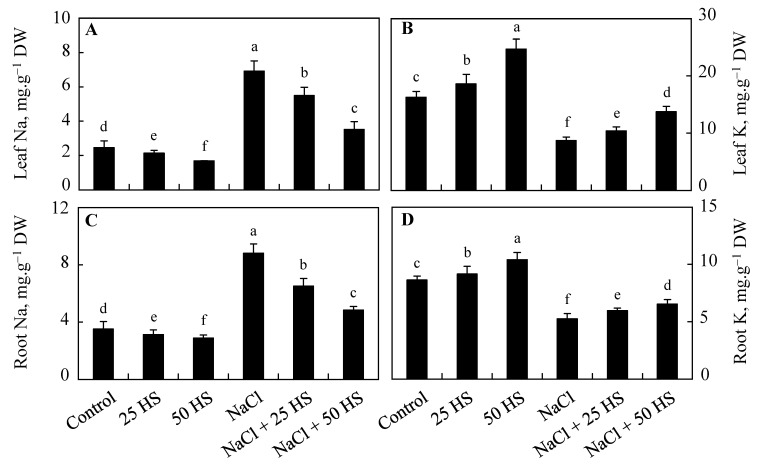
Effect of the exogenous supplementation of hydrogen sulfide (20 and 50 µM HS) on the (**A**) leaf sodium, (**B**) leaf potassium, (**C**) root sodium, and (**D**) root potassium content of wheat (*Tritium aestivum* L.) under salinity (100 mM NaCl) stress. The mean (±SE) of three replicates is given and bars with different letters are significantly different at *p* < 0.05.

Contrary to the control, the content of Na in roots and leaves increased by 151.42% and 180.16%, respectively, while K decreased by 39.39% and 46.49% in NaCl-treated plants. The content of Na decreased by 11.14% and 13.76% in roots and leaves, respectively due to 20 µM HS supplementation, and by 31.17% and 18.00%, respectively, due to 50 µM HS supplementation. However, K exhibited an increase of 14.57% in the leaves and an increase of 6.14% in the roots due to 20 µM HS, and respective increases of 51.90% and 20.50% due to 50 µM HS. HS treatment applied to NaCl-treated plants reduced the accumulation of Na and mitigated the decline in K. Dramatic declines of 45.11% (root) and 49.13% (leaf) in Na were observed in plants treated with NaCl + 50 µM HS over NaCl counterparts. Relative to NaCl-treated plants, K saw declines of 24.85% and 57.93% in roots and leaves, respectively, due to NaCl + 50 µM HS (Figure 9).

### 2.10. Effect of NaCl and HS on Endogenous HS and Nitric Oxide

**Figure 10:**
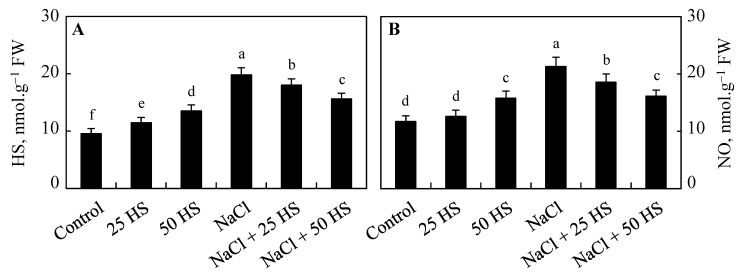
Effect of the exogenous supplementation of hydrogen sulfide (20 and 50 µM HS) on the endogenous content of (**A**) hydrogen sulfide (HS) and (**B**) nitric oxide (NO) of wheat (*Tritium aestivum* L.) under salinity (100 mM NaCl) stress. The mean (±SE) of three replicates is given and bars with different letters are significantly different at *p* < 0.05.

Endogenous concentrations of HS and nitric oxide (NO) saw a significant increase due to NaCl; however, exogenous HS treatment applied to NaCl-stressed counterparts resulted in a decline in the concentration of HS and NO. HS and NO saw increases of 107.98% and 82.05%, respectively, over the control due to NaCl. However, exogenous HS treatment applied to NaCl caused increases of 89.07% and 58.97% in plants treated with NaCl + 20 µM and increases of 63.86% and 37.60% in plants treated with NaCl + 50 µM. Under normal growth conditions, relative to the control plants, HS and NO increased by 19.85% and 7.69%, respectively, due to 20 µM HS and they increased by 41.38% and 35.04%, respectively, due to 50 µM HS (Figure 10A,B).

## 3. Discussion

Salinity stress imparts damaging effects on the normal growth and development of plants, causing considerable yield losses. From time to time, different management strategies have been devised and implemented to protect the crop plants from salinity-induced alterations in normal growth and metabolism. In the present study, the exogenous supplementation of HS was tested against the harmful influence of salinity in wheat. It was observed that salinity stress reduced growth in terms of the height, fresh weight, and dry weight of plants. However, HS treatment proved to be effective in considerably mitigating damage. A decline in growth due to salinity stress has been reported in many crop species [2,3,9,10,22,23]. The excess availability of salts can hamper growth by impeding the functioning of a cell cycle, hence causing significant declines in cellular division and tissue proliferation [24]. Salinity stress was found to reduce turgor and water uptake, and also induce osmotic and ionic stress [25]. However, HS supplementation increased the growth of wheat and also significantly alleviated the reduction. In support of our previous results, Chen et al. [26] (barley), Mostofa et al. [13] (rice), Jiang et al. [27] (cucumber), and Dawood et al. [28] (artichoke) have also reported significant results on alleviating salinity-induced declines in growth and biomass due to HS supplementation. Increased growth due to HS can be attributed to the improved uptake of essential ions (like K) and the selective absorption of Na concomitants with increased photosynthesis and other metabolic processes [8]. Previously, HS supplementation has been reported to facilitate the selective absorption of Na by regulating transport proteins, like PM ATPase and Na/H antiporters [26,27]. In addition, HS potentiates the uptake of beneficial elements, like K, by improving the high-affinity K uptake system and the inwardly rectifying the K channel, thereby reducing the Na/K ratio to prevent an ionic imbalance within cells [27]. Increasing the uptake of K benefits plants in several ways because of its active involvement in several plant processes, like enzyme functioning, photosynthesis, stress tolerance, etc. [5]. 

Moreover, the supplementation of HS at both concentrations increased photosynthetic pigments. Salinity stress brings down the synthesis of chlorophylls by inhibiting the activity of enzymes that mediate chlorophyll synthesis and reduce the uptake of mineral ions (like Mg) [23]. Stresses trigger the degradation of chlorophyll by activating chlorophyllase functioning [29]. The supplementation of HS may enhance the functioning of the chlorophyll biosynthesis cycle, and research in this direction can be fruitful. Previously, increased chlorophyll synthesis via HS treatment in *Spinacia oleracea* [30] and cucumber [27] has been reported. Increased carotenoid synthesis due to HS treatment helps to protect the photosynthetic system by mediating the quenching and scavenging of ROS and triplet-state chlorophylls, harvesting light and dissipating harmful excess energy [31]. Plants treated with exogenous HS exhibit significant improvements in stomatal and non-stomatal photosynthetic parameters under both normal and stress conditions [8].

The exposure of wheat plants to NaCl leads to significant improvements in oxidative stress attributes, like H_2_O_2_, MG, and lipid peroxidation. However, HS-supplemented seedlings caused a reduced accumulation of H_2_O_2_ and MG, thereby resulting in a considerable decline in lipid peroxidation with a concomitant increase in the MSI. In corroboration with these results, increased H_2_O_2_, MG, and lipid peroxidation has been reported by Ahanger et al. [9,10], Hasanuzzaman et al. [32], and Dawood et al. [28]. At optimal concentrations, H_2_O_2_ and MG are key in stress sensing and signaling; however, they impart damaging effects on growth and metabolism at higher concentrations [11,33]. Stress-induced increases in ROS can damage the membrane structure and alter its functioning, and HS supplementation can reduce ROS, as reflected in their declined lipid peroxidation and increased MSI. Reduced ROS due to HS treatment has been reported in rice [13], wheat [32], and artichoke [28].

The supplementation of HS improved the activity of antioxidant and glyoxylase I enzymes. Improved antioxidant functioning lowers the concentration of toxic radicals, therefore protecting cellular structures and their functioning [5,6]. Superoxide dismutase acts specifically on superoxide, while H_2_O_2_ is neutralized either by CAT or an intriguing ascorbate–glutathione cycle [5,6,9]. Improved antioxidant system functioning under salinity stress has been reported earlier [2,13,28]. In salt-stressed wheat, Ahanger et al. [9] demonstrated the mitigation of the oxidative effects of toxic radicals on photosynthesis due to increased antioxidant functioning. The improved functioning of the antioxidant system benefits plants by maintaining a redox status, thereby protecting enzymes, photosynthesis, and yield [4]. In artichoke, HS treatment increased the antioxidant activity, resulting in a significant decline in oxidative stress parameters [28]. In addition, an exogenous HS-mediated enhancement in antioxidant functioning has been reported to protect carbohydrate metabolism and photosynthesis under heat stress [8]. The improved functioning of the ascorbate–glutathione cycle protects plants from stresses by lowering H_2_O_2_ and maintaining NADP/NADPH for a continued electron transport system [5]. The supplementation of HS alleviated a decline in ascorbic acid under salt stress. Ascorbic acid and GSH are the key antioxidant molecules and redox components that play active roles in stress tolerance [34,35], and an increase in their concentration due to HS treatment may be contributed to salinity tolerance by maintaining redox homoeostasis and the activity of APX and GR. Glyoxylase I is a key enzyme in the glyoxylase cycle that acts to scavenge toxic methylglyoxal levels. Increased glyoxylase cycle activity due to HS treatment has also been reported in rice, resulting in significant decline in methylglyoxal content [13]. Maintaining lower concentrations of methylglyoxal prevents cells from cytotoxic effects, hence resulting in the maintenance of growth and metabolism [11].

Wheat plants exposed to salt stress caused a significant increase in proline and glycine betaine, which were further increased following exogenous HS treatment. Osmoprotectants play a very precise and important role in the stress tolerance of plants as they maintain water content, scavenge ROS, protect enzyme structure and functioning, maintain a redox state, and mediate stress signaling [36,37]. Increases in proline and glycine betaine in NaCl-stressed plants have also been reported by Khan et al. [38], Ahanger et al. [9,10], Dawood et al. [28], and Kumar et al. [39]. The greater synthesis of osmoprotectants results from modulations in the activity of their biosynthetic enzymes [2,37]. Hydrogen-sulfide-mediated increases in glycine betaine and proline have also been reported under different stresses [13,21,28,40].

Phenols play an important role in plant stress tolerance by regulating growth, membrane integrity, ROS metabolism, stress sensing, and response elicitation [41]. Salinity stress has been found to increase phenols, and similar results have been reported by Ahanger et al. [9] and Soliman et al. [1]. HS treatment caused an increase in phenols and phenylalanine ammonia-lyase activity under no-stress and salt stress conditions. Dawood et al. [28] have also demonstrated the increased activity of phenylalanine ammonia-lyase due to HS treatment, resulting in increased phenol accumulation under saline–alkaline stress conditions. Maintaining higher concentrations of secondary metabolites is directly influenced by phenylalanine ammonia-lyase functioning, and, in this study, it was obvious that HS treatment upregulated the activity of phenylalanine ammonia-lyase and the content of phenols. Exogenous HS-mediated increases in the functioning of enzymes that regulate secondary metabolite accumulation significantly contribute to oxidative stress alleviation and growth maintenance under stress conditions [21]. In addition, nitrate reductase activity was significantly increased due to HS treatment. Salinity significantly reduced the activity of nitrate reductase [9,10] and alleviated the decline in nitrate reductase due to exogenous HS, though this has not been previously reported. The reduced decline in nitrate reductase activity due to HS in arsenic-stressed pea plants has been reported by Singh et al. [19].

It was interesting to observe a significant modulation in the endogenous concentrations of HS and NO. Earlier increased concentrations of endogenous HS have been reported under waterlogging [40], heat stress [8], and salinity [13]. Salinity increases NO concentration [10]. The supplementation of HS resulted in a significant increase in endogenous NO concentrations; however, both HS and NO levels tended to be lower when HS was supplied to NaCl-stressed plants. Similar effects of applied HS on NO concentrations have been reported in heat-stressed wheat [8]. It seems that the influence of HS on stress tolerance regulation is dependent on the maintenance of NO levels, as has been reported by Singh et al. [19] under arsenic stress conditions. The expression of genes responsible for HS synthesis is triggered by stress [42]. Further, HS is considered to be a novel downstream signal molecule in NO-mediated stress tolerance [43], and further studies are required in this direction.

## 4. Material and Methods

### 4.1. Experimental Design, Growth Conditions, and Treatments

Uniform healthy seeds of wheat (*Triticum aestivum* L.) were sterilized with 0.01% HgCl_2_ for 5 min. Sterilized seeds were repeatedly washed with distilled water and were sown in earthen pots. Pots were filled with washed sand and were saturated with 300 mL full-strength Hoagland nutrient solution. After germination, the seedlings were thinned to ten per pot and were allowed to grow for two weeks normally. Thereafter, the pots were divided into two groups and one group was exposed to salinity stress by applying 100 mM NaCl using a modified Hoagland nutrient solution. To each group, the hydrogen sulfide (HS; in the form of NaHS) at 20 and 50 µM concentrations was also applied at the time of salt stress through the root medium. A freshly prepared nutrient solution was consistently applied. Overall treatments were as follows: (a) control (normal Hoagland), (b) 20 µM HS, (c) 50 µM HS, (d) 100 mM NaCl, and (e) NaCl + 20 µM HS and NaCl + 50 µM HS. Nutrient solution with and without NaCl and HS was applied on every alternate day for another four weeks. Six-week-old plants (four weeks after treatments) and seedlings were harvested, and different parameters (including morphological, enzyme activity, osmolytes, and mineral ions) were estimated. The protocols used are discussed below.

### 4.2. Growth Parameters

The height of the plants was determined using a scale. To measure the shoot fresh weight, the whole of the upper shoot part was taken and the fresh weight was recorded. Thereafter, the same shoot tissue was dried in an oven for 72 h at 60 °C to determine the shoot dry weight.

### 4.3. Measurement of Chlorophyll and Carotenoid Content

Chlorophyll a, chlorophyll b, total chlorophyll, and carotenoids were extracted by homogenizing the fresh leaves in 80% acetone, and the absorbance of the supernatant was taken at 645, 663, and 480 nm [44].

### 4.4. Measurement of Hydrogen Peroxide, Methylglyoxal, Lipid Peroxidation, and the Membrane Stability Index (MSI)

Velikova et al.’s [45] method was adopted for H_2_O_2_ measurement purposes. Briefly, after being extracted in trichloro acetic acid (TCA), potassium phosphate buffer (pH 7.0) and potassium iodide were added to the extract. Absorbance was taken at 390 nm. Lipid peroxidation was measured following Heath and Packer’s instructions [46]. Briefly, after the extraction of tissue in TCA, thiobarbituric acid was added for extraction and the mixture was then boiled at 95 °C. Absorbance was taken at 532 and 600 nm. Methylglyoxal (MG) content was extracted in perchloric acid (5%). After decolorizing with charcoal, supernatant, sodium dihydrogen phosphate, and N-acetyl-L-cysteine were mixed. After 10 min, the samples were read at 288 nm [47]. Sairam’s [48] method was used to measure the MSI and calculation was completed using the following formula:

MSI (%) = {1 − (EC1/EC2)} × 100

### 4.5. Determination of Proline and Glycine Betaine

Proline was extracted from dry tissue in sulphosalicylic acid and determined by reacting the extract with ninhydrin reagent. The optical density was recorded at 520 nm [49]. Glycine betaine was estimated following Grieve and Grattan’s [50] method in dry tissue. After extraction, the samples were diluted using 2N H_2_SO_4_. To known volume of diluted aliquot was added to the cold KI-I2 solution and the resultant was centrifuged for 15 min at 10,000× *g*. Thereafter, 1,2-dichloroethane was added to dissolve the periodide crystals. Optical density was read at 365 nm.

### 4.6. Assay of Antioxidant Enzymes and Ascorbate and Reduced Glutathione

To assay the superoxide dismutase, Bayer and Fridovich’s [51] method was followed, while Aebi’s [52] method was adapted for catalase determination. Nakano and Asada’s [53] method was used for ascorbate peroxidase, and Foyer and Halliwell’s [54] glutathione reductase method was also followed. However, ascorbate was estimated in line with Mukherjee and Choudhuri’s [55] method, and glutathione was reduced according to Ellman’s [56] method.

### 4.7. Activity of Glyoxalase I and Nitrate Reductase

Glyoxylase I (EC: 4.4.1.5) activity was assayed following Hasanuzzaman et al.’s [32] method in an assay mixture containing 100 mM GSH, 100 mM phosphate buffer, 16 mM MgSO_4_, and distilled water, and 35 mM methylglyoxal was then added to initiate the reaction. A 240 nm increase in optical density was noticed at 2 min. The activity of nitrate reductase was measured in accordance with Jaworski’s [57] method, and the absorbance was taken at 540 nm.

### 4.8. Determination of Total Phenols and Activity of Phenylalanine Ammonia-Lyase

Singleton and Rossi’s [58] method was employed for phenol estimation purposes. The activity of phenylalanine ammonia-lyase (PAL) was measured in fresh tissue and absorbance was taken at 290 nm [59].

### 4.9. Estimation of Na and K

The contents of Na and K were determined in acid-digested samples using a flame photometer.

### 4.10. Estimation of Hydrogen Sulfide and Nitric Oxide

To measure HS, fresh leaves were macerated in 20 mM Tris-HCl buffer (pH 6.8) containing 10 mM ethylene diamine tetraacetic acid, and the supernatant was mixed with 1% (*w*/*v*) zinc acetate. After 30 min, dimethyl-p-phenylenediamine (prepared in HCl) and ferric chloride (prepared in HCl) were added. The optical density was taken at 670 nm [60]. The content of NO was determined by reacting the extract with Griess reagent in accordance with Zhou et al.’s [61] method. The optical density was recorded at 540 nm.

### 4.11. Statistical Analysis

The data are the mean (±SE) of three replicates. Duncan’s multiple-range test was used to test the significance, and the least significant difference (LSD) was calculated at *p* < 0.05.

## 5. Conclusions

Salinity significantly reduced the growth of wheat by inducing oxidative damage through the excessive accumulation of toxic radicals, thereby causing membrane damage. The supplementation of HS affectively assuaged the damaging effects of salinity which can be attributed to the increased functioning of the antioxidant system and glyoxylase I activity. In addition, increased osmoprotectants and significant modulations in the endogenous concentrations of HS and NO due to HS supplementation can play a significant role in salinity tolerance. The upregulation of the antioxidant system, glyoxylase I activity, osmoprotection accumulation, and the selective uptake of Na in HS-treated plants potentially contributed to tolerance against salinity due to HS.

## Data Availability

Not applicable.
